# Physical Activity Reduces Metabolic Risk via Iron Metabolism: Cross-National Evidence Using the Triglyceride–Glucose Index

**DOI:** 10.3390/metabo14120651

**Published:** 2024-11-23

**Authors:** Zikang Hao, Xinmeng Guo, Yitao Wang, Guang Yang

**Affiliations:** 1 School of Physical Education, Shandong University, Jinan 250061, China; 2 Laboratory of Exercise Science, Ocean University of China, Qingdao 261000, China; 3 Kunshan Hospital of Traditional Chinese Medicine, Nanjing University of Traditional Chinese Medicine, Kunshan 215300, China; 4 School of Integrative Medicine, Nanjing University of Chinese Medicine, Nanjing 210023, China

**Keywords:** physical activity, triglyceride–glucose index, iron metabolism, metabolic health, lifestyle

## Abstract

**Purpose:** Studies suggest that the triglyceride–glucose index (TyG) is a novel and comprehensive marker of metabolic health. While most research indicates that increased physical activity (PA) is linked to improved metabolic health, some studies argue that the previous markers may not fully capture this relationship. This study uses TyG as a marker of metabolic health to examine the association between PA and TyG. **Methods:** Data are from cross-sectional surveys in three large population studies in China and the United States: CHARLS, CHNS, and NHANES. Regression models were applied to analyze the relationship between PA and TyG, with covariates adjusted in a stepwise manner. Stratified analysis was used to explore this relationship among different population groups, and, since it has been suggested that iron metabolism plays an important role in metabolic health, it was used as a mediating variable to construct a mediation model for analysis and discussion. **Results:** Higher PA was significantly associated with lower TyG levels across all three databases (*p* < 0.001), and this relationship remained robust after full adjustment for covariates. This negative association was more pronounced in older males (over 45 years). Iron metabolism also mediated this relationship, with mediation proportions ranging from 10% to 12.5%. **Conclusions:** There is a significant inverse association between PA and TyG, suggesting a link between increased PA and metabolic health, with iron metabolism moderating this relationship, especially among older males.

## 1. Introduction

The global incidence of chronic diseases related to metabolic abnormalities continues to rise, posing a significant burden on public health systems worldwide. Data indicate that, since 1975, the global obesity rate has nearly tripled; by 2016, more than 1.9 billion adults (aged 18 and over) were considered overweight, with over 650 million classified as obese [1]. The prevalence of metabolic syndrome (MeTS) among adults globally is estimated to be approximately 20–30% [2], and the number of individuals with type 2 diabetes mellitus (T2DM) is projected to reach around 700 million by 2045 [3]. Moreover, metabolic abnormalities are closely linked to the development of cardiovascular diseases, partly due to chronic inflammation and endothelial dysfunction caused by metabolic disturbances, which promote the progression of atherosclerosis [4]. Cardiovascular diseases account for over 17 million deaths annually, representing 31% of all global deaths [5]. These data suggest that metabolic abnormalities and their complications have become pressing global public health issues that require urgent attention.

Given the severe health problems associated with metabolic abnormalities, developing precise and convenient assessment tools is particularly important. Among the newly developed metabolic health risk assessment tools, the triglyceride–glucose (TyG) index has emerged as a sensitive and convenient biomarker. By combining triglyceride (TG) and fasting plasma glucose (FPG) levels, the TyG index can reflect an individual’s overall metabolic status and is considered a predictor of metabolic health [6]. Studies have shown that the TyG index is closely associated with insulin resistance (IR) and effectively predicts metabolic-related risks such as obesity, hyperlipidemia, and cardiovascular diseases. Compared with traditional IR detection methods like the homeostasis model assessment of insulin resistance (HOMA-IR), the TyG index demonstrates advantages in reflecting the breadth of metabolic health and predicting MeTS risk and has been widely used in epidemiological and clinical research. The current method of assessing IR using the HOMA-IR index is not applicable to insulin-treated diabetic patients because of the potential for interference with exogenous insulin, and it is not available in most parts of the Global South. IR is a well-recognized marker of metabolic disorders and systemic inflammation, which is not only an important risk factor for CVD but also leads to a poor prognosis. Current studies have demonstrated that the TyG index can be a reliable and convenient alternative to IR and that this index can be optimally used for risk stratification and outcome prediction of CVD, especially in developing countries [7].

Physical activity (PA), as an important lifestyle intervention, is generally believed to have positive effects on improving metabolic health, enhancing insulin sensitivity, and reducing the risk of metabolic abnormalities [8]. However, some studies have pointed out inconsistencies in the effectiveness of PA on improving metabolic risk, which may be related to limitations in assessment methods or differences in study populations [9]. This uncertainty underscores the necessity of introducing a more comprehensive metabolic health indicator to verify the role of PA. As an index which comprehensively reflects metabolic health, the TyG index can compensate for the shortcomings of previous PA studies in assessment methods. Therefore, systematically exploring the relationship between PA and the TyG index can help us fully understand the impact of PA on metabolic health and provide scientific evidence for early intervention.

Additionally, iron metabolism is considered to play a potential role in the development of metabolic abnormalities. The excessive accumulation of iron in the body can lead to oxidative stress, thereby interfering with insulin function [10]. Serum ferritin and transferrin saturation are key indicators reflecting iron metabolism status [11], and their abnormal levels are closely associated with the occurrence of IR and MeTS [12]. Some natural products, such as quercetin, may improve metabolic brain diseases, such as diabetic cognitive decline, by attenuating iron death, and these studies highlight how iron metabolic homeostasis can be helpful in maintaining metabolic health [13,14,15]. Studies have found that PA has certain antioxidant effects and can mitigate metabolic abnormalities caused by oxidative stress [16]. Interestingly, a study looking at exosomes found that exosomes in the plasma of young people in an active physical state improved iron death in the brain of older people [17]. Additionally, previous studies have suggested that skeletal muscle is no longer a separate motor organ but also a secretory organ, secreting substances such as muscle factors and exosomes which exert a cross-organ health-protective effect. These support the potential role of PA in improving metabolic health by regulating iron metabolism [18]. Therefore, we hypothesize that iron metabolism may mediate the relationship between PA and the TyG index. Understanding the mediating effect of iron metabolism can help clarify the potential mechanisms through which PA improves metabolic health by regulating oxidative stress in the body.

Lifestyle differences across different regions and cultural backgrounds may introduce biases in the study of the impact on metabolic health [19,20]. Variations in ethnicity, dietary habits, and environmental factors contribute to heterogeneity in the factors influencing metabolic health in global populations. Previous studies have mostly focused on surveys within a single region, lacking cross-national and cross-regional joint research [21,22,23], which weakens the universality and comprehensiveness of research findings.

Based on the above, this study aims to utilize data from China and the United States—two countries with large populations and significant cultural differences—employing three large public databases (CHARLS, NHANES, and CHNSto) to conduct a cross-national joint analysis. Using the TyG index as a comprehensive metabolic health assessment tool, we aim to systematically reveal the role of PA in overall metabolic health and its potential value in reducing metabolic risks. Additionally, this study will explore the potential mediating role of iron metabolism in the relationship between PA and the TyG index, providing scientific evidence for developing effective prevention and intervention strategies.

## 2. Methods

### 2.1. Study Design and Population

Data were obtained from three large public databases: the China Health and Retirement Longitudinal Study (CHARLS), the National Health and Nutrition Examination Survey (NHANES) of the United States, and the China Health and Nutrition Survey (CHNS). These databases provide extensive health information covering diverse geographical and cultural backgrounds.

In brief, CHARLS is a nationally representative longitudinal survey that began in 2011, encompassing approximately 17,000 respondents from 28 provinces, municipalities, and autonomous regions in China [24]. CHNS is an ongoing, prospective open-cohort study initiated in 1989, involving about 30,000 participants from 15 provinces, representing roughly 47% of China’s population [25]. NHANES is a continuous cross-sectional survey led by the Centers for Disease Control and Prevention (CDC) in the United States, employing a complex multistage stratified probability sampling design. Each survey cycle spans two years and has about 10,000 participants, sampled from multiple counties and cities across all 50 states and the District of Columbia [26]. Detailed study designs can be accessed from the respective official websites.

Consistent inclusion and exclusion criteria were applied across all databases. The inclusion criteria were the following: adults aged 20 years and above; complete PA data; available fasting plasma glucose (FPG) and triglyceride (TG) levels required to calculate the TyG index; measurements of iron metabolism indicators (such as serum iron and transferrin saturation); and complete demographic and health-related covariate information. The exclusion criteria included the following: pregnant women; individuals with missing key variable data; those diagnosed with major diseases affecting iron metabolism or insulin resistance (e.g., diabetes, severe hepatic or renal insufficiency, hematological diseases); and individuals with abnormal dietary energy intake (determined based on gender and physiological needs within a reasonable range).

All data used in this study were publicly accessible and had received ethical approval from relevant institutions. CHARLS was approved by the Institutional Review Board of Peking University, with all participants providing informed consent. The NHANES study was approved by the National Center for Health Statistics (NCHS) Ethics Review Board, and all participants provided written informed consent. The CHNS study received approval from the Institutional Review Boards of the University of North Carolina at Chapel Hill and the National Institute for Nutrition and Health, Chinese Center for Disease Control and Prevention, with all participants providing written informed consent. This study strictly adhered to the ethical principles of the Declaration of Helsinki. Data are publicly available through the respective official websites: CHARLS (http://charls.pku.edu.cn (accessed on 4 October 2024)), NHANES (https://www.cdc.gov/nchs/nhanes/index.htm (accessed on 4 October 2024)), and CHNS (http://www.cpc.unc.edu/projects/china (accessed on 4 October 2024)).

### 2.2. Independent Variable

The independent variable was the participants’ total PA over one week, encompassing three types: occupational PA, transportation PA, and leisure-time PA. Occupational PA referred to physical activities performed during daily work; transportation PA included activities like walking or cycling during commuting; and leisure-time PA involved exercise or sports activities undertaken for fitness purposes. In this study, we looked at the original questionnaires from the three databases, and the questions we referred back to were very similar, all based on self-reported PA over the course of a week, and included three main elements: the number of days per week on which light/moderate/heavy leisure/occupational/transportation activities (walking, cycling, etc.) were undertaken, and the time spent each day completing the above PA.

To standardize data from different databases, we calculated each participant’s total PA according to the International Physical Activity Questionnaire-Short Form (IPAQ-SF) calculation method. The specific calculation was as follows:

Total MET-mins/week = Walking MET-mins/week + Moderate-intensity MET-mins/week + Vigorous-intensity MET-mins/week

We adhered to the IPAQ standard guidelines for classifying activity intensity and assigning Metabolic Equivalent of Task (MET) values, ensuring comparability and consistency in PA calculations across databases [27,28].

### 2.3. Dependent Variable

The dependent variable was the TyG index, a composite indicator reflecting individual insulin resistance and overall metabolic health status. The TyG index combines fasting plasma glucose (FPG) and fasting triglyceride (TG) levels to effectively assess insulin resistance and is widely used in epidemiological studies and clinical practice [6]. The TyG index was calculated using the following formula:

TyG index = ln[(TG (mg/dL) × FPG (mg/dL)/2]

In CHARLS, FPG and TG were measured after the participants had fasted for at least 8 h, using the glucose oxidase–peroxidase method and the glycerol kinase enzymatic method, respectively. In CHNS, participants fasted for 8–12 h before examination; FPG was measured using the glucose oxidase phenol 4-aminoantipyrine peroxidase (GOD-PAP) method, and TG was measured using the glycerol phosphate oxidase–PAP method. In NHANES, participants fasted for at least 9 h before examination; FPG was measured using the hexokinase method, and TG was measured using an enzymatic assay. Although slight differences existed in measurement methods across the databases, they all employed internationally recognized standard methods with rigorous quality control measures, ensuring data reliability and comparability.

### 2.4. Mediating Variables

The mediating variables were commonly used indicators representing iron metabolism status: serum iron, transferrin, and transferrin saturation. FET reflects immediately available iron levels in the blood, and TFR, as the primary iron carrier, regulates iron distribution within the body [29]. These indicators collectively maintain the dynamic balance of iron in the body, ensuring normal physiological functions [30].

In CHNS, we used FET and TRF; in NHANES, we used FET and TRF. FET was measured using the ferrozine colorimetric method, while TRF was measured using immunoturbidimetry. Participants undergoing these measurements were required to fast for at least 8 h.

### 2.5. Covariates

To control for potential confounders and improve the accuracy of the results, the covariates below were included.

The following demographic factors were included: age (continuous variable, in years); gender (male or female, categorical variable); race/ethnicity (applicable to NHANES), including non-Hispanic White, non-Hispanic Black, Mexican American, and other ethnicities; and education level categorized by highest education attained (no formal education, primary school, middle school, high school and above).

The following lifestyle factors were considered: smoking status (never smokers, former smokers, current smokers); and alcohol consumption categorized by frequency and amount (non-drinkers, moderate drinkers, heavy drinkers).

The following health factors were also included: medical history including hypertension, cardiovascular diseases, and diabetes (included as categorical variables).

### 2.6. Statistical Analysis

Firstly, linear regression models were used to evaluate the association between PA and the TyG index. Three models were constructed: Model 1 was the unadjusted model analyzing the direct relationship between PA and the TyG index. Model 2 was adjusted for demographic variables, including age, gender, race/ethnicity, education level, and household income. Model 3 was further adjusted for health-related factors such as smoking status, alcohol consumption, body mass index (BMI), and waist circumference.

Next, to explore how different population characteristics influenced the PA and TyG index relationship, stratified (subgroup) analyses were conducted. Participants were categorized by age (>45 years vs. ≤45 years), gender (male vs. female), and PA intensity (PA > 600 MET-mins/week vs. PA ≤ 600 MET-mins/week). Linear regression analyses were repeated within each subgroup.

Then, to assess the mediating effect of iron metabolism indicators between PA and the TyG index, mediation analyses were performed. Serum iron and transferrin saturation were selected as mediators. The mediation analysis followed these steps: The mediation model evaluated the effect of PA on iron metabolism indicators, adjusting for covariates. The outcome model assessed the effects of PA and iron metabolism indicators on the TyG index, adjusting for the same covariates. Significance testing used the bootstrap method (1000 resamples) to calculate indirect effects, direct effects, and total effects, evaluating the significance of the mediation effect [31].

In processing NHANES data, given their complex sampling design, weighted linear regression models were employed. The ‘survey’ package in R was used to adjust for sampling weights, stratification, and clustering [32]. Covariate selection in all models was based on potential confounders, including demographic and health-related factors, to reduce bias.

Lastly, to ensure robustness, all continuous variables were tested for normality before model fitting. Variables with skewed distributions were log-transformed to meet the assumptions of linear regression. Categorical variables were included as dummy variables. Results were expressed as regression coefficients (β) with 95% confidence intervals (CIs). All statistical analyses were performed using R software (version 4.4.1), utilizing packages such as ‘survey’, ‘mediation’, and ‘tidyr’. Two-sided tests were conducted, with a significance level set at *p* < 0.05.

## 3. Results

### 3.1. Study Design and Participant Selection

**Figure 1:**
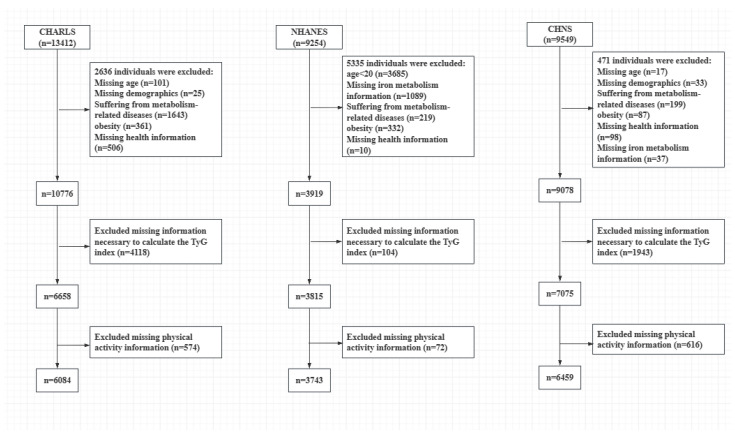
Flowchart of participant selection from the databases.

This study utilized waves from three databases that included physiological and biochemical indicators. Specifically, the CHARLS database used data from the 2015–2016 wave (totaling 13,412 individuals), NHANES used data from the 2017–2018 cycle (totaling 9254 individuals), and CHNS used data from the 2009 wave (totaling 9549 individuals). Based on the predefined inclusion and exclusion criteria, the final sample sizes were 6084 participants from CHARLS, 3743 from NHANES, and 6459 from CHNS. The participant selection flowchart is presented in Figure 1. The characteristics of the participants included in this study from each database are summarized in Table 1.

### 3.2. Negative Correlation Between PA and TyG Index

As shown in Table 2, a significant negative correlation between PA levels and the TyG index was observed across all databases.

CHARLS: In Model 1, for every increase of 1000 MET-minutes/week in PA, the TyG index decreased by 0.006 units (β = −0.006, *p* < 0.001). This relationship remained consistent after adjusting for confounding factors. In Model 2, the TyG index decreased by 0.005 units (β = −0.005, *p* < 0.001), and, in Model 3, it decreased by 0.004 units (β = −0.004, *p* < 0.001).

CHNS: Similarly, in Model 1, every 1000 MET-minutes/week increase in PA was associated with a 0.013-unit decrease in the TyG index (β = −0.013, *p* < 0.001). After adjusting for covariates, the results remained robust. In Model 2, the TyG index decreased by 0.010 units (β = −0.010, *p* < 0.001), and, in Model 3, it decreased by 0.009 units (β = −0.009, *p* < 0.001). These findings indicate that, in large Chinese populations, higher PA levels are associated with lower TyG index values.

NHANES: A similar negative correlation was found. In Model 1, each additional 1000 MET-minutes/week in PA corresponded to a 0.020-unit decrease in the TyG index (β = −0.020, *p* = 0.0014). After adjusting for confounders, this relationship persisted. In Model 2, the TyG index decreased by 0.021 units (β = −0.021, *p* < 0.001), and, in Model 3, it decreased by 0.018 units (β = −0.018, *p* = 0.008).

### 3.3. Stratified Analysis of the Negative Correlation Between PA and TyG Index

**Figure 2:**
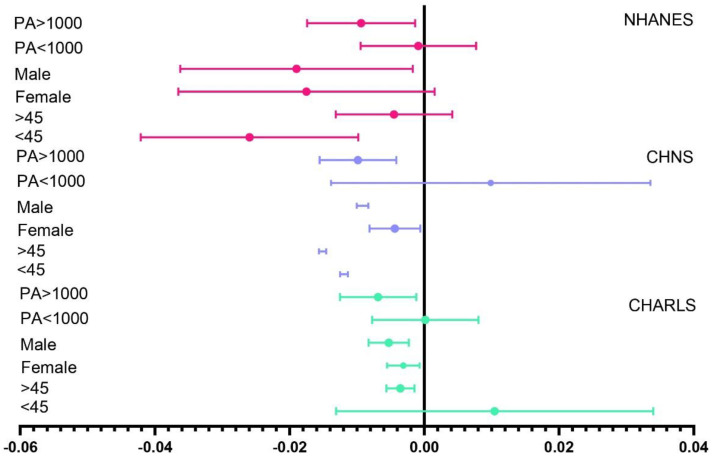
Stratified analysis of the inverse relationship between PA and TyG index. The horizontal coordinate represents the effect size. The colours represent different databases, green for CHARLS, blue for CHNS and pink for NHANES.

As illustrated in Figure 2, we conducted stratified analyses based on age (>45 years), PA levels (>1000 MET-minutes/week), and gender.

CHARLS: For participants younger than 45 years, every 1000 MET-minutes/week increase in PA was associated with a non-significant increase of 0.006 units in the TyG index (β = 0.006, *p* = 0.45). For participants older than 45 years, each 1000 MET-minutes/week increase in PA led to a significant decrease of 0.004 units in the TyG index (β = −0.004, *p* < 0.001). Among females, the TyG index decreased by 0.003 units per 1000 MET-minutes/week increase in PA, but this was not statistically significant (β = −0.003, *p* = 0.061). Among males, there was a significant decrease of 0.005 units in the TyG index per 1000 MET-minutes/week increase in PA (β = −0.005, *p* < 0.001).

Only participants with total weekly PA > 1000 MET-minutes showed a significant inverse relationship; the TyG index decreased by 0.008 units per 1000 MET-minutes/week increase in PA (β = −0.008, *p* < 0.001).

CHNS: Participants younger than 45 years showed a significant decrease of 0.010 units in the TyG index per 1000 MET-minutes/week increase in PA (β = −0.010, *p* < 0.001). Participants older than 45 years had a decrease of 0.020 units in the TyG index per 1000 MET-minutes/week increase in PA (β = −0.020, *p* < 0.001). Among females, the TyG index decreased by 0.008 units per 1000 MET-minutes/week increase in PA (β = −0.008, *p* < 0.001). Among males, the decrease was 0.010 units (β = −0.010, *p* < 0.001). Again, only participants with total weekly PA > 1000 MET-minutes showed a significant inverse relationship; the TyG index decreased by 0.009 units per 1000 MET-minutes/week increase in PA (β = −0.009, *p* < 0.001).

NHANES: Participants younger than 45 years showed a significant decrease of 0.020 units in the TyG index per 1000 MET-minutes/week increase in PA (β = −0.020, *p* = 0.03). For those older than 45 years, the TyG index decreased by 0.004 units per 1000 MET-minutes/week increase in PA, but this was not statistically significant (β = −0.004, *p* = 0.358). Among females, the TyG index decreased by 0.020 units per 1000 MET-minutes/week increase in PA, which approached significance (β = −0.020, *p* = 0.09). Among males, the decrease was significant at 0.010 units per 1000 MET-minutes/week increase in PA (β = −0.010, *p* = 0.0492). Only participants with total weekly PA > 1000 MET-minutes showed a significant inverse relationship; the TyG index decreased by 0.008 units per 1000 MET-minutes/week increase in PA (β = −0.008, *p* = 0.045).

### 3.4. Mediation Effect of Iron Metabolism on the Negative Correlation Between PA and TyG Index

**Figure 3:**
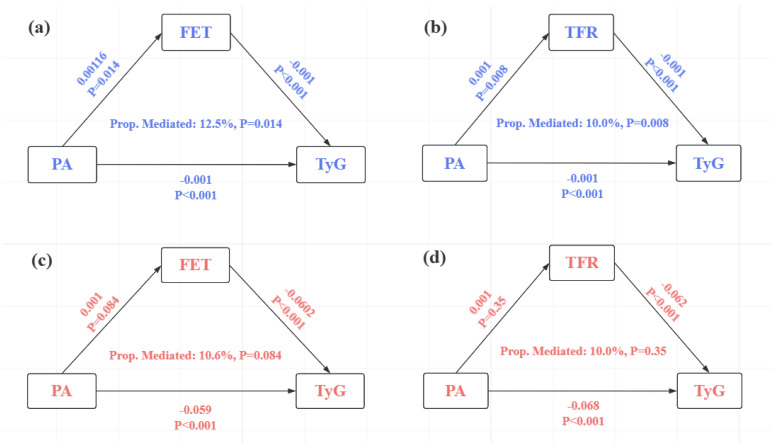
Mediation models of iron metabolism mediating the inverse relationship between PA and TyG index: (**a**) CHNS dataset with FET as the mediator; (**b**) CHNS dataset with TFR as the mediator; (**c**) NHANES dataset with FET as the mediator; and (**d**) NHANES dataset with TFR as the mediator. The left line of the triangle implies the effect of PA on iron metabolism metrics, the right line represents the effect of iron metabolism metrics on TyG, the lower line represents the effect of PA on TyG, and the value in the middle of the triangle represents the mediating share of iron metabolism metrics in the effect of PA on TyG. Red represents CHNS, blue represents NHANES, and arrows represent path coefficients.

The results of the mediation model analysis are shown in Figure 3. Both iron metabolism metrics were found to be significantly involved in the side effects of PA on TyG in the CHNS dataset, and the results were statistically significant, with a moderating effect of 12.5% for FET and 10.0% for TFR. In the NHANES dataset, although a moderating effect of FET as a mediator was also found, the effect was marginal and did not reach significance. On the other hand, although TFR may also have a mediating role, it did not contribute significantly.

## 4. Discussion

This study systematically explored the association between PA and the TyG index using large-scale population databases from China (CHARLS and CHNS) and the United States (NHANES). Additionally, we evaluated the mediating role of iron metabolism in this relationship. Our results demonstrated that increased PA levels were significantly associated with lower TyG index values, and this association remained robust after adjusting for confounding factors. Mediation analysis further revealed that iron metabolism indicators partially mediated the relationship between PA and the TyG index.

### 4.1. Analysis of Negative Correlation Between PA and TyG Index

The TyG index, as an emerging metabolic health assessment tool, has garnered widespread attention in recent years. Compared to traditional insulin resistance evaluation methods like HOMA-IR, the TyG index offers higher sensitivity and specificity, is simpler to calculate, and is cost-effective [6]. Studies have shown that the TyG index is closely associated with insulin resistance and is linked to the onset and progression of various metabolic diseases, including type 2 diabetes [33], metabolic syndrome [34], cardiovascular diseases [35], and non-alcoholic fatty liver disease [36]. Moreover, the TyG index can predict the risk of atherosclerosis [37], coronary artery calcification [38], and cardiovascular events [39], making it a reliable indicator of an individual’s overall metabolic status.

In our study, we adopted the TyG index as a tool to assess metabolic health, compensating for the limitations of single indicators in previous research and providing a more comprehensive reflection of individual metabolic conditions. By analyzing data from large populations, we found that increased PA levels significantly reduced the TyG index, further confirming the critical role of PA in improving metabolic health. This finding has significant public health implications, emphasizing the importance of PA as a lifestyle intervention and supporting its application in strategies to prevent metabolic risks.

From a physiological perspective, PA may influence the TyG index through multiple pathways. First, PA can enhance glucose uptake by muscles, improve insulin sensitivity, and lower fasting blood glucose levels [40]. Second, PA helps regulate lipid metabolism, reducing plasma triglyceride concentrations [41]. Additionally, PA can decrease visceral fat accumulation, improve adipose tissue function, and reduce the occurrence of insulin resistance [42]. These mechanisms collectively lead to a reduction in the TyG index, reflecting improvements in metabolic health.

### 4.2. Analysis of PA and TyG Index’s Negative Correlation in Different Populations

Given that different populations exhibit variations in fat distribution, hormone levels, and metabolic rates [43,44], we conducted stratified analyses. The results indicated that the negative correlation between PA and the TyG index was more pronounced in men and middle-aged and elderly individuals (>45 years).

In men, the positive impact of PA on metabolic health may be more evident. Men typically have higher muscle mass and basal metabolic rates; therefore, PA’s effects on energy and lipid metabolism are more significant [45]. Moreover, men are more likely to engage in high-intensity physical activities, potentially leading to greater metabolic benefits [46]. Studies have shown that after increasing PA, men experience greater improvements in insulin sensitivity and lipid metabolism [47], possibly due to higher testosterone levels, promoting muscle growth and fat oxidation [48].

In middle-aged and elderly populations, the negative correlation between PA and the TyG index was more robust. As age increases, metabolic function declines, insulin sensitivity decreases, and metabolic risks rise [49]. PA can alleviate age-related muscle loss (sarcopenia), enhance muscle glucose uptake, increase metabolic rate, and consequently improve metabolic health [8].

Our study also found that the negative correlation between PA and the TyG index was significant only when PA levels were high (total weekly PA > 1000 MET-minutes). This suggests that achieving a certain intensity and duration of PA is crucial for eliciting improvements in metabolic health. The World Health Organization recommends that adults engage in at least 150 min of moderate-intensity or 75 min of vigorous-intensity aerobic activity per week [50]. Research has confirmed that high levels of PA are necessary to significantly impact lipid metabolism and insulin sensitivity [9].

Notably, in the U.S. young population (≤45 years), the negative correlation between PA and the TyG index was more significant. This may be related to differences in lifestyle and metabolic characteristics between Chinese and American populations. Young Americans may be more susceptible to obesity and metabolic abnormalities, making PA interventions more effective in this group [51]. In contrast, Chinese middle-aged and elderly individuals are more prone to metabolic syndrome, and the benefits of PA are more pronounced in this population [52].

### 4.3. Analysis of the Mediating Role of Iron Metabolism in the Relationship Between PA and the TyG Index

Iron metabolism abnormalities play a crucial role in the development of metabolic disorders, particularly the impact of iron overload-induced oxidative stress on insulin resistance and metabolic syndrome [53]. Excessive iron promotes the production of reactive oxygen species (ROS), leading to increased oxidative stress levels, which damage insulin secretion and function [54,55]. Oxidative stress can also cause lipid peroxidation and protein damage, accelerating metabolic dysfunction [56].

In this study, we selected serum iron and transferrin as representative indicators of iron metabolism. Serum iron reflects the level of bioavailable iron in the blood, while transferrin is responsible for iron transport and is a vital component of iron metabolism [57]. These indicators can represent an individual’s iron metabolic status and help assess the relationship between iron metabolism and metabolic health.

Extensive research has confirmed the close association between iron metabolism abnormalities and metabolic disorders. High levels of serum iron and ferritin are linked to an increased risk of type 2 diabetes [58], and iron overload can lead to insulin resistance and glucose metabolism disorders [59]. These findings suggest that iron metabolism abnormalities may promote the occurrence of metabolic abnormalities by inducing oxidative stress and inflammatory responses [60].

Interestingly, some studies have found that PA can modulate iron metabolism and alleviate metabolic diseases. For example, in mice in which obesity was induced by a high-fat diet, moderate exercise reduced hepatic iron content, decreased oxidative stress levels, and improved insulin resistance [61]. PA may increase iron consumption and utilization, reducing excessive iron accumulation in the body and lowering oxidative stress [62]. PA can affect the expression and activity of proteins related to iron metabolism, such as TfR1, divalent metal ion transporter 1 (DMT1), and membrane iron transport protein 1 (FPN1), which are involved in iron uptake and release [63]. By regulating these proteins, PA may help maintain homeostasis of iron metabolism and positively impact metabolic health [64]. PA activates autophagy not only in contracted muscles but also in non-contracted tissues like the liver. Autophagy is an intracellular clean-up process that breaks down and recycles damaged or non-essential intracellular structures, which is essential for maintaining cellular and overall metabolic health [65].

Physiologically, PA regulates iron metabolism through multiple pathways, influencing metabolic health. First, PA increases muscle demand for iron, promotes erythropoiesis, and enhances iron utilization. Second, PA can modulate the expression of iron-regulating hormones (such as hepcidin), promoting iron excretion [66]. Additionally, PA enhances the activity of antioxidant enzymes, reducing oxidative stress-induced cellular damage [67].

Our mediation analysis supports the partial mediating role of iron metabolism in the relationship between PA and the TyG index. This finding provides new insights into the mechanisms by which PA improves metabolic health and underscores the importance of regulating iron metabolism in the prevention and intervention of metabolic disorders.

A very noteworthy point regarding the differences in iron metabolism between the Chinese and American populations is that the results leading to the differences may be caused by a variety of factors. For example, genetic factors, such as the C282Y and H63D mutations in the HFE gene, have a large difference in distribution between races, and these mutations affect the normal activity of hepcidin, which, in turn, affects iron metabolism. In the Chinese population, the distribution of these mutations differs from that in the European and American populations [68,69]. In addition to this, dietary habits may also have an impact. Dietary habits and lifestyles in different regions can lead to differences in intestinal flora, which can affect iron metabolism. For example, in Western populations, diets containing iron-fortified cereals reduced the abundance of *Bifidobacterium* spp, *Lactobacillus* spp, and *Rhodobacter* spp, compared to diets containing less iron-rich meat [70].

### 4.4. Strengths, Limitations, and Public Health Implications

This study has several strengths. First, we utilized large, representative population databases from China and the United States, enhancing the generalizability of our findings. Second, we employed the TyG index, a sensitive and convenient metabolic health assessment tool, providing a comprehensive evaluation of metabolic status. Third, we explored the mediating role of iron metabolism, deepening our understanding of the mechanisms by which PA affects metabolic health.

However, there are limitations to our study. First, the cross-sectional design cannot establish causality; longitudinal studies are needed for verification. Second, PA data were primarily self-reported, which may have introduced information bias; objective measurement methods are recommended. Third, the iron metabolism indicators were limited and may not have fully captured the complexity of iron metabolism. Finally, despite adjusting for multiple confounding factors, residual confounding may still exist, such as the actual amounts of dietary iron consumed by the participants and genetic tendencies towards iron status.

Our findings have significant public health implications. They emphasize the critical role of PA in improving metabolic health, supporting its inclusion in strategies to prevent metabolic abnormalities. Revealing the mediating role of iron metabolism between PA and metabolic health provides new perspectives for intervention measures. The cross-national results support the universality of PA’s benefits across different populations, holding broad policy and practical significance.

Future studies should focus on the following areas: first, conducting longitudinal research to verify the causal relationship between PA and the TyG index; second, delving deeper into the mechanisms of how iron metabolism influences the impact of PA on metabolic health, particularly at the molecular and genetic levels; and third, evaluating the effects of different types and intensities of PA on metabolic health to develop more precise intervention strategies.

## 5. Conclusions

Increased PA is associated with improved metabolic health, as indicated by lower TyG index values, and iron metabolism partially mediates this relationship. These findings highlight the importance of promoting PA in preventing metabolic abnormalities and suggest that regulating iron metabolism could enhance the beneficial effects of PA. In addition, more scientific methods are needed to thoroughly verify this preliminary finding.

## Figures and Tables

**Table 1:** The characteristics of the participants included in this study.

	CNHS	NHANES	CHARLS
**Count**	6084	3743	6459
**Age**	50.422 (14.579)	51.504 (17.813)	59.982 (10.245)
**Height**	160.797 (8.171)	Use BMI 29.852 (7.396)	166.218 (11.372)
**Weight**	60.198 (9.962)		63.221 (15.853)
**Gender**			
Male	2683 (44.1%)	1681 (44.9%)	3042 (47.1%)
Female	3401 (55.9%)	2062 (55.1%)	3417 (52.9%)
**Edu**			
1	3218 (52.9%)	468 (12.5%)	3675 (56.9%)
2	1722 (28.3%)	939 (25.1%)	1931 (29.9%)
3	961 (15.8%)	1482 (39.6%)	672 (10.4%)
4	183 (3.0%)	853 (22.8%)	181 (2.8%)
**Smoke**			
Yes	1843 (30.3%)	1804 (48.2%)	2590 (40.1%)
No	4241 (69.7%)	1939 (51.8%)	3869 (59.9%)
**Drink**			
Yes	2002 (32.9%)	2699 (72.1%)	3979 (61.6%)
No	4082 (67.1%)	1044 (27.9%)	2480 (38.4%)
**Rural**			
Yes	4088 (67.2%)	N/A	4179 (64.7%)
No	1996 (32.8%)	N/A	2280 (35.3%)
**TyG**	8.397 (1.454)	8.553 (0.699)	8.718 (0.644)
**TFR**	287.586 (55.141)	27.262 (11.652)	N/A
**FET**	138.394 (86.575)	185.963 (35.868)	N/A
**PA**	2472.178 (125.068)	1303.742 (71.882)	2418.007 (82.189)

**Table 2:** Stepwise regression results of PA and TyG index.

	Model 1		Model 2		Model 3	
	β	*p*	β	*p*	β	*p*
**CHARLS**	−0.006	<0.001	−0.005	<0.001	−0.004	<0.001
**CHNS**	−0.013	<0.001	−0.010	<0.001	−0.009	<0.001
**NAHNES**	−0.020	0.001	−0.021	<0.001	−0.018	0.008

Model 1: simple regression analysis of PA and TyG index; Model 2: regression analysis adjusted for demographic covariates; Model 3: regression analysis fully adjusted for demographic and lifestyle covariates; and β: regression coefficient indicating effect size.

## Data Availability

The raw data used in this study can be found in the Methodology Section.
